# Could non-humans have Traditional Ecological Knowledge? And if so, what should we do about it?

**DOI:** 10.1186/s13002-026-00877-6

**Published:** 2026-04-05

**Authors:** Elodie Freymann, Fabien Schultz, Daniel Redfearn

**Affiliations:** 1 Brown University, Institute at Brown for Environment and Society, Rhode Island, USA; 2 Bernhard Nocht Institute for Tropical Medicine (BNITM), Ethnopharmacology and Zoopharmacognosy, Hamburg, Germany; 3 Neubrandenburg University of Applied Sciences, Department of Agriculture and Food Sciences, Neubrandenburg, Germany; 4 Belfast Health and Social Care Trust, Department of Psychiatry, Belfast, UK

**Keywords:** Non-Human Traditional Ecological Knowledge, Interspecies ethics, Traditional Medicinal Knowledge, Zoopharmacognosy, Ecological culture

## Abstract

Traditional Ecological Knowledge (TEK) has historically been understood as uniquely human. However, emerging evidence from elephants, cetaceans, chimpanzees and other intelligent species suggests that some animal communities maintain and transmit ecological knowledge across generations in ways comparable to human TEK. Here, we propose reframing these knowledge systems as Non-Human Traditional Ecological Knowledge (NHTEK), emphasizing the intergenerational transmission of ecological knowledge in non-human species. We extend this discussion to the emerging field of Zoopharmacognosy (the study of animal medication), where preliminary findings raise the possibility that certain species, in particular wild chimpanzee (*Pan troglodytes*), may possess TEK related to medicinal resource-use. This possibility forces us to ask: *are we ethically prepared for such a finding*? Recognizing the existence of NHTEK challenges us to reconsider how we value non-human knowledge, how we protect it against commercial exploitation, and how conservation, intellectual property, and environmental governance might adapt in response.

## Science highlights

We argue that acknowledging the existence of non-human traditional ecological knowledge can shift anthropocentric perspectives and inform conservation policies aimed at protecting ecological cultures and relevant resources.

## Introduction

In major intergovernmental science-policy frameworks, Traditional Ecological Knowledge (TEK) is broadly understood as place-based ecological knowledge, practices, and beliefs acquired through direct contact with the environment and transmitted across many generations within indigenous and local communities (e.g., [1]). It is this policy-oriented definition that we adopt for the purposes of this paper. However, TEK is not a monolithic concept, and we acknowledge that this framing captures only one dimension of a far richer and more contested body of thought. Scholars have long pushed back on singular and limiting definitions of TEK [2], and emphasized that these systems are more than just transferable bodies of knowledge. Instead, many have argued that TEK is fundamentally embedded in relationships, responsibilities, and ways of knowing that are inseparable from the communities that hold them (e.g., [3], [4]). Specifically, several indigenous scholars have stressed that TEK must be understood in relation to sovereignty, self-determination, and the enduring legacies of colonialism, dimensions that policy-oriented definitions risk obscuring [4, 5]. It is precisely because TEK carries this political and relational weight that extending its terminology to non-human systems, as we do here, requires careful justification, to which we return in Sect. "Extending TEK terminology: justification and considerations".

Within policy-related frameworks, TEK has long been considered a uniquely human phenomenon [6–8]. However, we argue here that this understanding reflects an anthropocentric worldview that is increasingly challenged by research on animal cognition and culture. This paper introduces the novel concept of Non-Human Traditional Ecological Knowledge (NHTEK), with a particular focus on traditional medicinal knowledge in non-human animal populations, challenging our fundamental assumptions about what constitutes traditional knowledge and who can possess it.

Famously, the commercial and academic exploitation of human TEK has caused past and present injustices [9]. Scientists have long neglected their ethical responsibilities towards indigenous communities, particularly in the fields of drug discovery and development [10, 11]. If non-human animals possess TEK systems, especially those with potential commercial applications, these systems could be vulnerable to similar patterns of exploitation. We ask here whether the time has come to acknowledge the existence of NHTEK and what our responsibilities are to protect it. This perspective introduces new directions for conservation biology, animal cognition research, drug discovery, and environmental law.

## Traditional Ecological Knowledge and its significance

Human TEK systems, which predate modern science, emerge from multi-generational lived experience within specific environments [1, 2, 12]. Unique to each community and ecosystem, they inform practices that enhance survival and adaptation within environments. In recent years, TEK has gained recognition not only within environmental research but also in major intergovernmental science-policy frameworks [13]. For example, Article 8 of the Convention on Biological Diversity calls for governments to “respect, preserve and maintain knowledge, innovations, and practices of indigenous and local communities embodying traditional lifestyles relevant for the conservation and sustainable use of biological diversity…” (United Nations, [14]). Organizations such as the Intergovernmental Panel on Climate Change (IPCC) and the Intergovernmental Science-Policy Platform on Biodiversity and Ecosystem Services (IPBES), have also sought to incorporate TEK alongside scientific data in their assessments and policy recommendations. But how does TEK relate to the broader concept of ecological cultures?

## Traditional Ecological Knowledge as a foundation of ecological cultures

Human cultures, broadly defined, are dynamic systems which encompass socially transmitted information, practices, and beliefs that shape how community members live and interact with each other and the world around them [15, 16]. When aspects of these cultural systems are organized around ecological relationships (including, but not limited to, foraging strategies, resource management, or medicinal plant use), they can be characterized as ecological cultures [1], the academic study of which is called Cultural Ecology [17]. To emerge, these ecological cultures require the input of ecological knowledge, or in other words, ecological information that has been internalized, processed, and applied through our understanding of, and experience with, the natural world (e.g., Nonaka and Takeuchi [18]. This ecological knowledge informs and governs our relationships with our physical environments, which we then enact and maintain through ecological-cultural behaviors (which we will call eco-cultural behaviors) and practices.

To sustain these ecological cultures across generations, mechanisms must exist for the transmission of ecological knowledge. Over time, as this knowledge is passed along through social learning processes and cultural transmission, ecological knowledge becomes “traditional”. Without the social transmission of TEK, which for example can include information on seasonal rain patterns, resource locations, or a plant’s medicinal properties, each generation would face the prohibitive costs of rediscovering essential survival information through individual trial-and-error [19]. It is this socially transmitted, intergenerationally stable TEK, therefore, that enables ecological cultures to persist [1, 20].

The relationship between TEK and culture, however, is not unidirectional. TEK not only drives ecological cultures by informing and enabling ecological practices, but it also becomes a product of these same cultures, embedded within the traditions and practices that are transmitted to subsequent generations. In this dynamic cycle, ecological knowledge (e.g., which plants are medicinal) informs practice (e.g., how to prepare and administer these medicinal plants), practice reinforces knowledge (e.g., successful medicinal treatments are repeated), and both knowledge and practice are then culturally transmitted to sustain adaptive strategies over time (e.g., medicinal plant knowledge and preparation methods are passed on to the next generation). This cyclical process transforms TEK from a driver of culture into a reproducible cultural product (e.g., [1, 21, 22]).

We argue, therefore, that the eco-cultural behaviors and practices that constitute multigenerational ecological cultures necessarily presuppose Traditional Ecological Knowledge. This framework for thinking about the relationship between ecological cultures and TEK has important implications for non-human species. If a species or non-human community demonstrates ecological culture, it follows that some form of TEK must also be present.

## From animal culture to Non-Human Traditional Ecological Knowledge

While TEK, up to this point, has been considered unique to humans, as our understanding of cognition and culture across the non-human animal kingdom expands, we are confronted with a new and provocative question: ***Can we extend the concept of Traditional Ecological Knowledge to non-human societies*?**

### Culture in the animal kingdom

Until the mid-20th century, culture was also considered uniquely human. This perspective began to shift with Japanese anthropologist Kinji Imanishi’s groundbreaking suggestion that if non-human individuals within a species could learn from one another, distinct groups might develop unique behavioral variations [23, 24]. Over time, as the behavioral repertoires of non-human animals became better understood, scientists began to find evidence of culture across diverse species. These discoveries revealed behavioral variation within species which could be attributed to neither genetic nor ecological variables (e.g., [25], [26]). Rather, evidence began to emerge to suggest that many of these behavioral traditions appeared to meet operational definitions of culture, demonstrating their ability to be socially transmitted between members of the same species or social group, and persist over time (e.g., chimpanzees: [26–28]; sparrows: [29]; Japanese macaques: [30]; bottlenose dolphins: [31]). While there have been many definitions of non-human culture proposed throughout the last few decades, today it is commonly defined as socially learned behaviors that persist over time, and which become characteristic of a local population (e.g., [32–34]).

### Non-human ecological knowledge systems

Most ethologists now acknowledge that many non-human animals possess complex cultures [35], but are any of these non-human cultures ecological? In other words, are any of these non-human cultural behaviors organized around or dependent on ecological relationships? If so, is there evidence that these ecological cultures are also guided by multi-generational, place-based ecological knowledge, which we will now refer to as Non-Human Traditional Ecological Knowledge (NHTEK)? The short answer is yes. Evidence from across the animal kingdom suggests that many non-human cultural practices may indeed meet this definition.

African elephants (*Loxodonta africana*) provide a compelling case for the existence of non-human ecological cultures, which are driven by NHTEK. These highly social mammals maintain and transmit detailed knowledge about the location and seasonal availability of water sources, mineral licks, and safe migration routes [36]. African elephants have also been shown to exhibit site-fidelity to resource locations regardless of resource quality [37]. Older matriarchs serve as notable repositories of this ecological knowledge, vertically transmitting specific information about resource locations and seasonal temporality across generations, though preferences are also likely reinforced through mixed-sex, multi-age group gatherings, which allow for observational learning opportunities [37]. The intergenerational transfer of ecological knowledge ensures community survival over time and allows elephants to navigate highly variable and sometimes harsh environments.

Bottlenose dolphins (*Tursiops sp.*) in Shark Bay, Western Australia, also exhibit ecological cultural behavior informed by knowledge of their local environment. These wild dolphins maintain a unique foraging strategy which exploits local prey distribution and habitat structure, utilizing sea sponges to protect their rostrums while searching for fish on the seafloor [31]. This hunting technique is adapted to incorporate characteristics of these sponges, as well as local prey behavior and environmental conditions, suggesting these practices are guided by specific ecological knowledge. Sponging behavior, which represents the first case of material culture in a marine mammal, has been shown to be transmitted vertically through the matriline [31].

Killer whales (*Orcinus orca*) similarly demonstrate cultural behaviors uniquely tuned to their environments, and which appear to be shaped by multi-generational knowledge of local ecosystems. For example, like dolphins, certain killer whale pods use hunting techniques tailored to the specific behaviors and distributions of local prey species, such as intentional beaching to capture seals or coordinated herding of fish schools [38, 39]. These techniques persist across generations through social learning, indicating that knowledge of prey behavior and environmental conditions is transmitted within pods.

Evidence of multi-generational eco-cultural practices can also be found in the nut-cracking behaviors of Western chimpanzees (*Pan troglodytes verus*). These practices demonstrate sophisticated ecological knowledge (as well as technological skill), including an understanding of nut availability, nut hardness, and optimal extraction strategies [40, 41]. This knowledge informs decisions on which nuts to target, which tools from the environment to select, and how to position nuts to maximize efficiency and safety. Furthermore, archaeological evidence from Côte d’Ivoire’s Taï Forest indicates that these behaviors (and therefore the ecological information governing them) have likely persisted for millennia [42]. Stone tools used for nut-cracking, dating back 4,300 years, exhibit characteristic wear patterns matching those produced by modern chimpanzees. The apparent continuation of these practices across ~200 generations lends further evidence that the ecological knowledge underlying nut-cracking is socially learned and culturally transmitted, constituting a form of NHTEK. Notably, similar archaeological evidence of primate stone tools has been found at a capuchin nut-cracking site in Serra da Capivara National Park Brazil [43, 44]. These tools suggest that nut-cracking, as a capuchin technological cultural tradition, has persisted at this site for millennia, though damage patterns demonstrate that food processing has likely shifted over time. The authors note that these shifts may represent adaptive responses to cultural variation in capuchin diets [43].

While NHTEK may lack the symbolic or linguistic dimensions of human TEK systems, we argue that it can be understood as a functional analogue: a socially transmitted repository of ecological information that enables adaptive interaction with the environment.

### Defining NHTEK: operational criteria and distinctions

We argue that NHTEK differs from other forms of non-human cultural information in its place-based ecological specificity, duration of persistence, and ability to specifically inform eco-cultural behaviors and cultures. To rigorously apply the concept of NHTEK in future research, we propose four operational criteria: **(1) Ecological Specificity**: The knowledge must pertain to specific environmental features, resources, or ecological relationships within the community’s habitat. **(2) Multi-generational Persistence**: The knowledge must be maintained across at least three generations, demonstrating temporal stability beyond individual or parent-offspring transmission. **(3) Social Transmission**: The knowledge must spread through social learning mechanisms (observation, teaching, imitation) rather than genetic inheritance alone. **(4) Cultural Influence**: The knowledge must demonstrably inform ecological cultures or eco-cultural behaviors.

### Distinguishing NHTEK from related phenomena

By our definition, ecological knowledge in non-human individuals obtained through individual learning or acquired spontaneously due to a genetic predisposition, while important, does not constitute NHTEK unless it is then spread and maintained through social transmission across several generations. A single elephant discovering a new water source through exploration represents individual learning; that knowledge becomes “traditional” only when transmitted to and maintained by subsequent generations through social mechanisms. Additionally, not all knowledge that is socially transmitted qualifies as NHTEK. The criterion of ecological specificity is crucial: behaviors must be governed by and encode information about ecological environmental properties, relationships, or dynamics. For example, socially learned social conventions in chimpanzees, such as group-specific hand-clasp grooming styles, represent cultural knowledge maintained across generations, yet are social rather than ecological in nature [45]. Similarly, the temporal requirement of multi-generational persistence distinguishes NHTEK from short-lived behavioral fads which may emerge and disappear within a group across one or two generations, e.g., tail walking in Indo-Pacific bottlenose dolphins (*Tursiops aduncus*) along the southern coast of Australia [46].

## Non-human medicinal knowledge

If, as we argue, non-human species possess systems of TEK, this raises further questions about the specific forms of ecological knowledge non-human communities may maintain and transmit. One particularly intriguing possibility is that some non-human species may possess TEK related to health maintenance and medicine. In humans, intergenerational health-related knowledge that is sustained by communities over time is often referred to as Traditional Medicinal Knowledge (TMK) [47]. This typically includes knowledge of medicinal plants and other naturally occurring resources, as well as their health-related applications. TMK systems, particularly those which involve the use of medicinal plants, are present across human populations worldwide. The World Health Organization (WHO) reports that plants serve as the sole source of primary healthcare for at least 80% of the world’s population ([48]; [49]). As of 2025, the Royal Botanic Gardens, Kew’s Medicinal Plant Names Services, contained more than 39,000 plant species with documented ethnomedicinal uses [50]. TMK has not only sustained communities for generations but has also become increasingly valuable in modern pharmaceutical research and development [51].

While researchers have yet to definitively demonstrate the presence of medicinal cultures in non-human animals, several studies in Zoopharmacognosy (the study of animal medication) suggest these cultures are likely present (e.g., [27, 28, 52]). Here, we provide a brief overview of this emerging field and highlight evidence suggesting that these behaviors may also be driven by underlying systems of NHTEK.

### The case for traditional medicinal knowledge in wild chimpanzees

Animals across the animal kingdom have demonstrated the ability to identify and utilize naturally occurring medicinal resources found within their environments (see [53] for review). These behaviors take many forms and can be observed across many species including, but not limited to: birds [54], butterflies [55]; bears [56], sheep [57], and chimpanzees [58]. While many advancements have been made in the field of zoopharmacognosy in the last few decades, a central question remains: *Do these medicinal behaviors stem from innate instincts*,* individual learning*,* or socially transmitted medicinal knowledge?* At present, there is still much debate over whether these non-human medicinal practices constitute medicinal cultures, but ongoing research is exploring this possibility.

Among non-human animals, chimpanzees represent a particularly well-documented species for investigating the presence of medicinal cultures. Wild chimpanzees have been shown to exhibit several medicinal behaviors which include the exploitation of naturally occurring resources in their environment (e.g., [59–61]). In recent years, several medicinal behaviors involving novel resources have been observed across certain chimpanzee groups. For example, researchers in Gabon documented chimpanzees catching insects and applying them to open wounds [62]. Due to the unambiguous context in which this behavior was observed, the authors propose that the application of these insects likely promotes healing or protects against infection. A similar behavior was recently observed in a different chimpanzee community in Kibale, Uganda [63], yet it remains notably absent from neighboring groups. The presence of this behavior in some communities but not others suggests that insect application could constitute a community-specific medicinal tradition, akin to other resource-related technological behaviors such as termite fishing [64] and moss-sponging [65], both of which are recognized as cultural traditions. First, however, the ecological explanation, that these behaviors reflect the availability of insects in some places but not others, must be ruled out.

Researchers have also hypothesized that certain medicinal behaviors in chimpanzees may be transmitted across generations through social learning rather than being genetically encoded [58, 66]. This hypothesis is supported by observations of individuals copying the medicinal behaviors of other group members [27, 28], as well as notable differences in the medicinal resource repertoires of neighboring chimpanzee communities that cannot be explained by genetic differences or ecological variation [52].

As the field of zoopharmacognosy develops and methods for studying animal behavior become more sophisticated, it is increasingly plausible that non-human animals will be found to possess medicinal cultures related to the use of specific medicinal resources within their environment. If proven, these medicinal cultures, which involve ecological relationships with naturally occurring resources, could be appropriately classified as ecological cultures. Consistent with our framework, the presence of these medicinal eco-cultures would therefore imply the presence of medicinal NHTEK, or what we could also refer to as Non-Human Traditional Medicinal Knowledge (NHTMK).

From a cognitive perspective, the presence and transmission of NHTMK is entirely plausible. Just as place-based NHTEK guides culturally transmitted ecological behaviors that rely on knowledge of the local environment (e.g., migration routes, feeding ecology, organic tool-use), so too could NHTMK drive and become a product of intelligent species’ medicinal cultures, provided this medicinal knowledge is socially transmitted and maintained across generations. We therefore argue that the cognitive capacities required to maintain systems of NHTMK are well within the abilities of chimpanzees, and other intelligent and cultural species.

## Ethical considerations and responsibilities arising from NHTEK

Ethically, acknowledging the existence of NHTEK challenges long-standing anthropocentric assumptions about whose knowledge is worthy of respect and protection. In human societies, intergenerational knowledge systems are increasingly safeguarded through intellectual property regimes (e.g., Treaty on Intellectual Property, Genetic Resources, and Associated Traditional Knowledge, 2024), biocultural protocols, and benefit-sharing agreements, such as the Nagoya Protocol [13]. If NHTEK systems play a similar role in supporting survival and adaptation in non-human communities, then humans may have an obligation to consider precautionary frameworks of protection that prevent the exploitation, disruption, or destruction of these knowledge systems.

The possible existence of NHTMK in chimpanzees provides a compelling illustration of why these ethical questions are critical to consider. Even without definitive proof that animals possess and maintain medicinal cultures, the possibility alone prompts preemptive consideration. If, for example, humans discover new medicinal resources by studying the multigenerational medicinal behaviors of chimpanzees and commercially exploit these resources for pharmaceutical development, should this constitute a form of biopiracy? Should corporate entities be held accountable for sharing the benefits and revenues derived from this discovery with the chimpanzees from whom this knowledge was extracted? It is important to highlight that were this knowledge to have originated from a human TMK system, the answer would be yes, not merely as an ethical position, but as a matter of established international law. What if, through the exploitation of non-human medicinal knowledge, a commercial actor curtailed a non-human community’s access to critical medicinal resources — should that community be granted legal standing to seek reparations? If so, what form would these reparations take, and who would be charged with representing “the human face” [67] of these non-human medicinal knowledge holders? Finally, what if humans and non-humans have overlapping traditional medicinal knowledge systems or share medicinal resources within a given environment? How would benefit-sharing mechanisms and reparations be structured to avoid reproducing the historical exploitation of Indigenous and local communities and their knowledge systems? Recognizing the existence of NHTEK forces us to confront the historical pattern in which human traditional knowledge has been appropriated without consent or protection (e.g., [68]) and consider whether similar exploitation of non-human knowledge systems should be permitted.

The broader implications of this acknowledgment extend to conservation initiatives. Programs geared at protecting habitats and protecting against population loss are necessary but insufficient if they ignore the social structures, learning pathways, and intergenerational knowledge systems that underpin adaptive behaviors [69]. The examples provided above of elephant migration, orca hunting, dolphin foraging, and chimpanzee tool-use, all represent eco-cultural behaviors that rely on socially transmitted ecological knowledge and which are all highly vulnerable to anthropogenic disruptions [70, 71]. Conservation strategies that neglect these dimensions risk eroding not only behavioral repertoires but also the NHTEK systems which sustain them. This perspective echoes recent calls from ethologists, conservationists, and IUCN working groups, such as the Working Group for Chimpanzee Cultures (WGCC), to put protections in place for preserving non-human cultures [69].

## Extending TEK terminology: justification and considerations

Some may question the need for the term NHTEK when concepts such as “ecological cultures” and “cultural knowledge” already exist in the animal behavior literature. Two key considerations justify this terminology. First, unlike the term “ecological cultures”, NHTEK treats transmitted ecological knowledge as the fundamental unit, distinguishing it from its behavioral or cultural expressions. Second, the term “cultural knowledge” lacks both ecological specificity and temporal depth, as it can encompass transient social fads rather than enduring, multigenerational, place-based knowledge systems.

Instead, we use the term NHTEK to parallel historical uses of TEK in policy contexts, emphasizing the functional equivalence of these systems in supporting adaptive responses to environmental challenges and their practical implications for conservation. This framing highlights an analytic analogy while also signaling future responsibility. Just as acknowledging TEK in science-policy frameworks has led to the creation of safeguards for human TEK, extending TEK to non-human systems implies the need for careful stewardship of these knowledge systems to protect them from anthropogenic extinction and/or deleterious exploitation. 

At the same time, we acknowledge that TEK, as conceptualized in anthropology and Indigenous studies, carries deep cultural, historical, and political significance tied to human communities, particularly Indigenous peoples whose knowledge systems have been systematically undervalued, appropriated, and exploited [4, 9, 10]. Our extension of this terminology to non-human species is not intended to diminish or appropriate these human knowledge systems, but rather to highlight fundamental parallels in how non-human ecological knowledge is generated, maintained, and transmitted across generations. We emphasize that recognizing NHTEK should complement, not compete with, efforts to protect human TEK. Both systems face similar threats, such as habitat destruction, cultural disruption, and commercial exploitation, and both deserve robust protections.

## Implications and conclusion

Here, we have argued for the recognition of Non-Human Traditional Ecological Knowledge (NHTEK) and for further exploration of the ethical and practical responsibilities that arise from this recognition. These responsibilities include developing new ethical frameworks to protect NHTEK from deleterious exploitation, safeguarding ecological resources that underpin these knowledge systems, and preserving opportunities for social transmission which enable intergenerational transfer of ecological knowledge.

Acknowledging the existence of NHTEK, however, is not solely about protection; it is also about advancing scientific frameworks and approaches to studying the non-human world. Non-human species possess deep ecological knowledge that humans have yet to fully comprehend. Engaging respectfully with these knowledge systems provides a unique opportunity to expand our own ecological understanding of the natural world and in doing so, improve conservation strategies.

In conclusion, the last few decades of animal behavior research have demonstrated that non-human species produce and maintain ecological cultures, which we argue are guided by traditional ecological knowledge systems. Classifying these knowledge systems as traditional, challenges anthropocentric assumptions, raises important ethical questions, and prompts new advancements in conservation, legal, and governance frameworks. By acknowledging the presence of these NHTEK systems, we can ensure the protection of this knowledge, promote increased respect for non-human communities, and benefit from the lessons they offer about survival and adaptation in complex and changing ecosystems.

## Data Availability

Not applicable.

## Abbreviations

TEK: Traditional Ecological Knowledge
NHTEK: Non-Human Traditional Ecological Knowledge
IPCC: Intergovernmental Panel on Climate Change
IPBES: Intergovernmental Science-Policy Platform on Biodiversity and Ecosystem Services
TMK: Traditional Medicinal Knowledge
WHO: World Health Organization
NHTMK: Non-Human Traditional Medicinal Knowledge
WGCC: Working Group for Chimpanzee Cultures
